# Genetically Engineered T Cells and Recombinant Antibodies to Target Intracellular Neoantigens: Current Status and Future Directions

**DOI:** 10.3390/ijms252413504

**Published:** 2024-12-17

**Authors:** Ana Maria Waaga-Gasser, Thomas Böldicke

**Affiliations:** 1 Renal Division, Department of Medicine, Brigham and Women’s Hospital, Harvard Medical School, Boston, MA 02115, USA; 2 Helmholtz Centre for Infection Research, 38124 Braunschweig, Germany

**Keywords:** neoantigens, TCR-like antibodies, intrabodies, bispecific antibody (CD3 × TCR, CD3 × TCR-like antibody), TCR-CARs, TCR-like CARs, T CARs, STARs, therapeutic mRNA, checkpoint blocking antibodies, CRISPR/Cas9-based genome editing

## Abstract

Recombinant antibodies and, more recently, T cell receptor (TCR)-engineered T cell therapies represent two immunological strategies that have come to the forefront of clinical interest for targeting intracellular neoantigens in benign and malignant diseases. T cell-based therapies targeting neoantigens use T cells expressing a recombinant complete TCR (TCR-T cell), a chimeric antigen receptor (CAR) with the variable domains of a neoepitope-reactive TCR as a binding domain (TCR-CAR-T cell) or a TCR-like antibody as a binding domain (TCR-like CAR-T cell). Furthermore, the synthetic T cell receptor and antigen receptor (STAR) and heterodimeric TCR-like CAR (T-CAR) are designed as a double-chain TCRαβ-based receptor with variable regions of immunoglobulin heavy and light chains (VH and VL) fused to TCR-Cα and TCR-Cβ, respectively, resulting in TCR signaling. In contrast to the use of recombinant T cells, anti-neopeptide MHC (pMHC) antibodies and intrabodies neutralizing intracellular neoantigens can be more easily applied to cancer patients. However, different limitations should be considered, such as the loss of neoantigens, the modification of antigen peptide presentation, tumor heterogenicity, and the immunosuppressive activity of the tumor environment. The simultaneous application of immune checkpoint blocking antibodies and of CRISPR/Cas9-based genome editing tools to engineer different recombinant T cells with enhanced therapeutic functions could make T cell therapies more efficient and could pave the way for its routine clinical application.

## 1. Introduction

Over the last two decades, cancer immunotherapy has been developed with substantial success demonstrating the prolonged survival of patients with rapidly fatal cancers. Antibody- and immune cell-based therapies are in the process of becoming first-line therapeutic steps in several hematological and solid cancers. The concept of cancer immunotherapy and particularly T cell-based strategies is based in part on the hypothesis that neoantigens become recognized and targeted by the immune system to prevent carcinogenesis, which resembles allograft rejection after organ transplantation [1]. Understanding antigen recognition and how to control underlying complex immune pathways seems to be critically important, with recent developments in TCR-like antibodies, bispecific antibodies, intrabodies and TCR-CAR strategies in tumor therapy [2,3,4,5]. Among the basic fundamentals, it should be recapitulated that CD4^+^ T cells recognize peptide antigens in the context of major histocompatibility complex (MHC) class II molecules and that they orchestrate the adaptive immune response while CD8^+^ T cells recognize antigens in combination with MHC class I molecules and are responsible for cytotoxic responses that are killing neoplastic or infected cells. Secondly, the T cell receptors on the surface of CD4 and CD8 T cells recognize antigens through a clone-specific pathway with highly polymorphic single α- and β-glycoprotein chains and non-polymorphic CD3 γ, δ, ε and ζ chains. This results in a broad repertoire of T cell clones with unique specificities through α- and β-chain gene segment rearrangements within the genome of the T cells. Positive and negative thymic selection enables a tolerant immune system against self-antigens (self-tolerance) while maintaining strong effectiveness against pathogens and tumor antigens. TCR clone-specific characterization and the timely recognition of occurring rearrangements of TCRs in response to new tumor antigens of interest seem therefore to be fundamentally important in the development of TCR- CAR, TCR-T cell therapies and TCR-like antibody repertoire development. Moreover, for effective T cell activation and proliferation, additional signaling is needed, described as a process of co-stimulation, which is required for the intracellular phosphorylation of early signal transduction within the T cells. The surface protein CD28 and its family are the most vigorous co-stimulatory receptors on T cells. B7-1 and B7-2 on antigen-presenting cells are ligands that become upregulated, enabling the binding of T cells in the context of Toll-like receptors, pathogens and likewise tumor neoantigen sensors. Surface molecules such as cytotoxic T lymphocyte-associated protein 4 (CTLA-4) and programmed cell death 1 (PD-1) act to diminish or inhibit hyperactivation during the immune response. Recently developed derivatives called checkpoint inhibitors are currently in clinical use for the blockade of such negative signaling events in cancer therapy. Notably, repeated low-dose and low-affinity stimulation can result in an exhausted phenotype of activated T cells in response to cancer development over time as reported for chronic infection or, in contrast, a subset of memory T cells. The latter are primed T cells that react more vigorously to the same antigen, which makes them important mediators of tumor immune recall responses. Technological advances in single-cell RNA sequencing together with transcriptomic, epigenomic and clonotypic changes in tumor-derived T cells make genetically engineered T cells and recombinant antibodies a successful immunotherapy. Particularly, well-established phage display technology makes it now possible to select TCR-like antibodies and nanobodies against any neoantigen [6].

**Figure 1:**
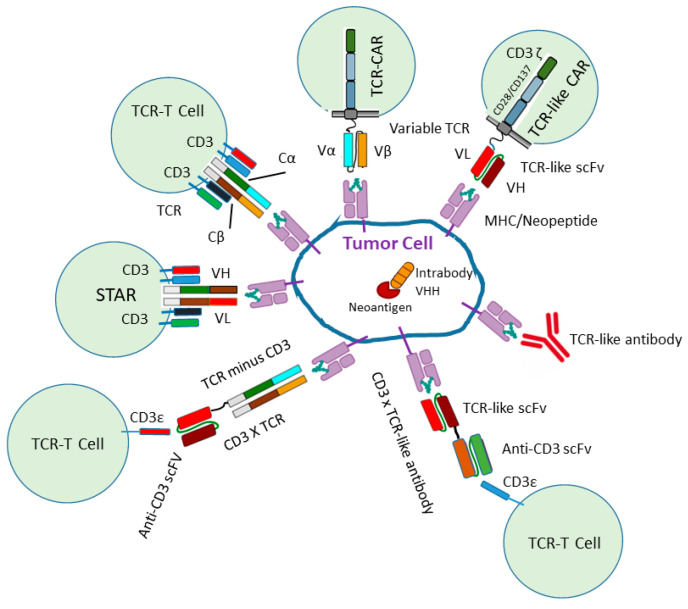
Neoepitope targeting with recombinant T cell receptors and recombinant antibodies. TCR-CARS target the neopeptide/MHC with the variable domains of a TCR, TCR-like CARs and TCR-like antibodies. TCR-engineered T cells express a new complete TCR on the cell surface and STARs express the variable regions of immunoglobulin heavy and light chains (VH and VL) fused to TCR-Cα and TCR-Cβ. Bispecific antibodies can target CD3ε and Neopeptide/MHC in the format soluble TCR × anti-CD3 or TCR-like antibody × CD3. TCR-like antibody can also be applied as a complete IgG antibody. In addition, intrabodies can neutralize the function of a neoantigen inside the cell.

Mutated neoantigens, which are specifically expressed inside tumor cells and not inside normal cells, represent an attractive target for cancer immunotherapy [7,8]. Four different strategies against such neoantigens are possible and currently under investigation: (i) knockout and knockdown techniques with CRISPR/Cas [9] and RNAi, respectively [10]; (ii) the functional inactivation of kinases by small molecule inhibitors [11]; (iii) targeting with recombinant T cells [5]; and (iv) targeting with TCR-like antibodies, bispecific antibodies and intrabodies [2,3,4]. Specific targeting of neoepitopes expressed through the major histocompatibility complex (MHC) as an MHC class I/peptide complex (pMHC) can be accomplished with different T cell approaches: autologous tumor-infiltrating lymphocytes expressing endogenous TCRs [12], TCR-engineered T cells [5,13,14,15,16], T cells expressing novel TCRs as the binding domain of the chimeric antigen receptor (TCR CARs) or a TCR-like antibody (TCR-like CARs) [17] (Figure 1).

**Figure 2:**
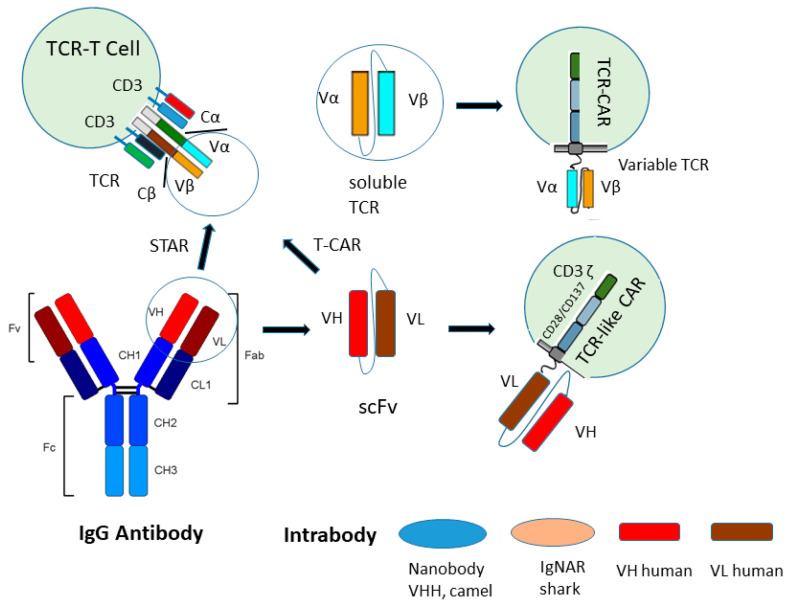
Transfer of variable regions of the TCR and IgG to TCR CAR, TCR-like CAR, STAR and T-CAR. In TCR-CAR, the TCR variable regions Vα and Vβ are linked with a flexible peptide linker and a transmembrane domain and fused to the chimeric antigen receptor with the costimulatory TCR domains. In STAR, the VH and VL domains of antibodies are fused to Cα and Cβ of a TCR. In T-CAR, a scFv (VH and VL linked with a flexible peptide linker) is fused to Cα or Cβ of TCR. In TCR-like CAR, a scFv is fused to a chimeric antigen receptor. Intrabodies are single-domain antibodies from camels, sharks or human VH or VL domains transferred via mRNA lipid nanoparticles or viral cell transduction into the nucleus or cytoplasm.

In addition, the synthetic T cell receptor, antigen receptor (STAR) and heterodimeric TCR-like CAR (T-CARS) are designed as a double-chain TCRαβ-based receptor with variable regions of immunoglobulin heavy and light chains (VH and VL) fused to TCR-Cα and TCR-Cβ, respectively, resulting in TCR signaling [18,19] (Figure 1 and Figure 2). 

For an efficient T cell activation of TCR CARs and TCR like CARs soluble TCRs and recombinant TCR-like antibody fragments are fused to a hinge or spacer peptide and a transmembrane domain followed by the intracellular T cell signaling domains, CD3ζ or CD3ε of the T cell receptor, and costimulatory domains such as CD28 or/ and 4-1BB leading to destruction of the tumor cells (Figure 1) (Figure 2). The enhanced T cell activation can subsequently lead to more efficient destruction of the tumor cells.

Two different types of neoantigens have been described in the past and should be distinguished in the context of T cell-based as well as antibody/intrabody-based therapies: Oncogenic mutations and highly recurrent gene fusions that can be localized in genome hotspots and that are shared among patients (so called ‘public’ neoantigens). These neoantigens contain the same genetic alterations and human leucocyte antigen expression patterns. In contrast, private neoantigens are more frequently identified in non-essential loci for tumorigenesis and metastasis formation, termed “passenger” mutations, and are specific for individual cancer patients [7,8]. Due to the absence of neoantigen expression in normal tissue, the low toxicity of drugs can be expected in non-affected cells [20]. Moreover, high-avidity T cells specific for these neoantigens are not eliminated during negative thymic selection and can be isolated not only from patient tumors but also from healthy donor peripheral blood [21].

For neopeptide identification, tumor-specific peptide sequences must be identified from next-generation sequencing data. Whole-genome sequencing (WGS), whole-exome sequencing (WES) and RNA sequencing are commonly used for that purpose [22].

**Figure 3:**
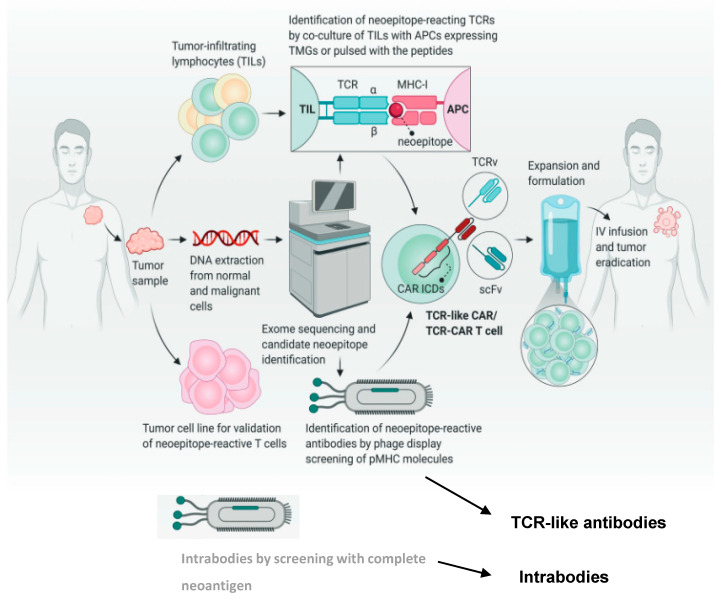
Current methodologies to identify neoepitopes and develop TCR-like CARs, TCR-CARs, TCR-like antibodies and intrabodies. Neoepitopes are identified by comparing whole genome sequencing and RNA sequencing of normal and tumor tissue. Neoepitope-reactive TCRs are then selected by co-culture of tumor-infiltrating lymphocytes (TILs) with APCs expressing identified tandem minigenes (TMGs) or neoantigen peptides. Characterized TCR variable domain (TCRv) or single-chain variable fragment (scFv) specific for neoepitope/MHCs are used in a CAR structure to produce TCR-CARs or TCR-like CARs, respectively. Modified T cells are then expanded and infused into the patient. CAR ICDs represent intracellular domains (ICDs). Furthermore, TCR-like antibodies and intrabodies can be selected with phage display by biopanning with neopeptide/MHCs or neoantigen, respectively. Figure 3 is a modification of Figure 2 in [17].

The alignment of the exosome DNA and RNA sequencing (RNA-seq) data of tumor cells and normal cells, including RNA-seq expression analysis, will lead to a growing set of tumor-specific neoantigen peptides [8,23]. In parallel, the human leukocyte antigens (HLA) can be determined via the Human Leukocyte Antigen Typing Algorithm [24] or predicted from single-cell transcriptome data [25]. Alternatively or in combination in silico sequence-based predictions, chromatography–mass spectrometry (LC-MS) can be used to directly identify the “immunopeptidome” or “ligandome” of tumor cells [23]. Finally, identified peptides are expressed as tandem mini-gene libraries or synthesized as long peptides, and patient T cells are tested regarding their capacity to recognize autologous antigen-presenting cells after transduction and protein transfection with the peptide candidates (Figure 3). Furthermore, peptide–MHC binding and stability have to be assessed with bioinformatics programs [7]. Recently, MHC anchor locations were demonstrated to guide neoantigen identification and prioritization [26], and a new platform was established for the identification of neoepitope–HLA pairs for neoantigens shared among patients [27].

For targeting neoantigen epitopes with TCRs, TCR-like CARs, STARs, T-CARs and TCR-like antibodies and bispecific antibodies, the appropriate TCR that binds a newly detected neopeptide/MHC must be identified. This can be performed by TCR sequencing of single-sorted neoepitope-reactive tumor-infiltrated T cells (Figure 3) [28] or by sorting neoantigen-positive single T cells from the peripheral blood of healthy donors [21,29,30]. Alternatively, a personalized CD8^+^ T cell library from cancer patients can be screened with predicted soluble fluorescence-labeled neoantigen–HLA complexes to isolate appropriate T cells for TCR sequencing [31]. These cells express patient-specific neoTCRs that target specific mutations of their cancer.

CAR-T cells recognize cell surface-expressed tumor-associated antigens and demonstrate great success in certain hematological cancers [5]. The five-year data with the first FDA-approved CAR-T cell therapy (tisagenlecleucel, Kymriah) for patients with relapsed or refractory B cell acute lymphoblastic lymphoma showed that 55% of patients were alive 5 years after treatment. https://www.novartis.com/news/media-releases/novartis-five-year-kymriah-data-show-durable-remission-and-long-term-survival-maintained-children-and-young-adults-advanced-b-cell-all, accessed on 14 December 2024 [32].

In Kymriah, the patient’s own T cells become reprogrammed with a chimeric antigen receptor (CAR) containing an anti-CD19 scFv and a 4-1BB costimulatory domain. In addition, the KYMRIAH CAR contains a CD3ζ intracellular signaling domain, which is critical for initiating T cell activation and antitumor activity. The anti-B cell maturation antigen (BCMA) CARs in Abcema contain an anti-BCMA single-chain variable fragment (scFv) coupled to the CD8α hinge and transmembrane domains, and the intracellular CD137 co-stimulatory (4-1BB) and CD3ζ chain signaling domains as well. In a phase 2 study of 140 patients with relapsed and refractory myeloma, idecabtagene vicleucel (Abcema) led to a response in almost 75% of affected patients [33].

Recently, CAR T cells against the T cell receptor beta-chain constant domain 1(TRBC1) have been applied in an international multicenter, single-arm phase 1/2 study in relapsed/refractory peripheral T cell lymphoma (NCT03590574). Ten patients were transduced with recombinant CAR T cells expressing the anti-TRBC1 scFv (JOVI-1) on the cell surface. A complete metabolic response was seen in four of six responding patients. In two patients, a partial response was observed. Among three patients receiving the highest dose level, two patients were in ongoing remission at 15 and 18 months, respectively, and received no further treatment. One patient receiving the lowest dose level achieved a complete metabolic response, relapsed at month two and died on study day 190.

In another approach, a drug-conjugated TCR-like antibody (Tesirine, SG3249) against TRBC1 was developed, showing that the antibody was internalized by the tumor cells and killed the cells in vitro and in vivo as demonstrated with immunodeficient mice injected with Jurkat TRBC1^+^ tumor cells. After treatment with the drug-conjugated antibody, tumor cells were undetectable and the cancer did not relapse. The authors also showed that CAR T cells expressing the anti-TRBC1 scFv (JOVI-1) on the cell surface were lost because they were killed by the patients’ normal T cells, leading to low efficiency [34,35].

A limited number of clinical trials are currently underway to investigate CAR-T cell therapies in patients with solid tumors of the breast (https://clinicaltrials.gov/study/NCT04650451), pancreas (https://clinicaltrials.gov/study/NCT03323944), lung, ovaries, (https://clinicaltrials.gov/study/NCT03054298) (URL (accessed on 15 December 2024)) and melanoma, demonstrating promising results [36]. However, one should refrain from prematurely drawing conclusions based on the small number of participating patients in these studies, as they have limited imaging and biopsy data. The phase 2 interim results from an ongoing NIH-funded trial with recombinant TCR T cells pointed to objective clinical responses using RECIST criteria, including the regression of metastases to the liver, lung and lymph nodes in three out of seven patients with metastatic, mismatch repair-proficient colorectal cancers who had progressive disease following multiple previous therapies [37], https://clinicaltrials.gov/study/NCT03412877 (URL (accessed on 15 December 2024)), Administration of Autologous T cells Genetically Engineered to Express T cell Receptors Reactive Against Neoantigens in People With Metastatic Cancer.

## 2. Targeting Mutation-Associated Neoantigens with TCR-T Cells, TCR-CARs, TCR-like CARs, T-CARs and STARs

### 2.1. TCR-T Cells

Mutation-associated neoantigens develop out of cancer-initiating genetic changes or from extensive genetic instability [38]. TP53, KRAS or PIK3CA are frequently mutated driver genes and examples of neoepitopes frequently found in tumor patients with specific HLA alleles [39]. TCR-T cells directed against TP53 mutations, for instance, are now the subject of clinical investigation in breast cancer, showing a time-limited response in a case with tumor progression after 6 months, relating to a loss of MHC class I expression [40,41]. The safety and tolerability of KRAS-G12D-specific TCR-T cells were tested in a patient with pancreatic cancer with tumor regression, no toxicity described after 6 months, and persisting TCR-engineered T cells in the circulation [42]. TCR-T cells directed against another mutated KRAS gene (KRASG12V) were likewise tested (NCT03190941). TCR-T cells engineered against specific mutated PIK3CA genes in HLA-A*03:01 patients demonstrated antitumor responses against tumors in preclinical murine models established with PIK3CA-mutated tumors but not wildtype PIK3CA tumors [43]. Another example is a TCR-engineered T cell therapy against the MAGE-A4-peptide–HLA complex that became recently FDA-approved in patients with rare soft tissue tumors [44,45].

These clinical data from the first TCR-T cell therapies in solid cancers are encouraging, although developing patient-individualized T cell therapies remains demanding in terms of laboratory and immunological/clinical expertise. Moreover, passenger mutations not involved in the carcinogenic pathway rather than commonly found mutation-associated neoantigens may soon become more attractive targets as a result of these first experiences. This is related to the complex process of identifying epitopes that are located within the mutated public gene areas and are suitable candidates considering the HLA-restricted antigen presentation. Interestingly, CRISPR gene editing techniques are now becoming ever more prevalent for the development of personalized TCR-T cells [31]. Neoantigen-specific TCRs were isolated from participating patients’ blood cells and cloned. The endogenous self-TCR genetic segments were deleted and the sequences encoding the selected neoantigen-specific TCRs were simultaneously inserted. After the administration of one to three different TCR-T cell therapies and the detection of the TCR-T cells migrating to the tumors, one-third of the patients developed a stable disease [31].

Moreover, the combination with an anti-PD-1 antibody as an add-on immune checkpoint inhibitor in this approach is now being tested in the clinic (NCT03970382 and NCT04520711). In this trial, neoantigen-reactive TCRs were isolated from tumor-infiltrating lymphocytes of patients with metastatic colorectal cancer and other gastrointestinal cancers, and the TCR α and β chains were incorporated into gamma retroviral vectors for the transduction of patients’ T cells. Target neoantigens were defined as mutated shared oncogenes, e.g., TP53, KRAS or oncoviral proteins. The transduced T cells were adoptively transferred into the patients after lymphodepleting chemotherapy. The administered T cell populations contained at least 50% TCR-transduced cells, and were described to be poly-functional in their ability to secrete IFNγ, GM-CSF, IL-2 and Granzyme B in response to mutant peptides compared with wild-type counterparts. Interestingly, TCR-transduced cells were detected in the peripheral blood of five patients at levels ≥10% of CD3^+^ cells 1 month after the adoptive cell transfer. One patient who responded to the therapy showed around 20% of CD3^+^ peripheral blood lymphocytes expressing transduced TCRs for more than 2 years after treatment.

This is in line with other reported results from individual TCR-T cell therapies, such as in a patient with chemorefractory metastatic cholangiocarcinoma and with a relatively pure population of neoantigen reactive T cells, which was grown from tumor-infiltrating T cells and mediated a near-complete regression of all metastatic disease, lasting more than 2.5 years [46].

The adoptive cell transfer of genetically modified peripheral blood lymphocytes to express personalized neoantigen-reactive TCRs can indeed mediate tumor regression in patients with metastatic cancers such as the one described with colorectal cancer (ClinicalTrials.gov registration: (NCT03412877) [37].

### 2.2. TCR-CARs and TCR-like CARs

In addition to T cells transfected with α and β TCR genes (TCR-T cells), neoantigen-specific TCR-CARs can be generated, expressing the variable domains of a TCR as the binding domain of the chimeric antigen receptor (Figure 1). TCR-CARs have been constructed by expressing the two extracellular domains of a TCR specific to MART-1 peptide 26–35 (EAAGIGILTV) and the TGFbR2 frameshift neoantigen peptide fused to CD28 and CD3ζ. TCR-CARs were able to redirect T cells, resulting in TCR-dependent killing in vitro [47].

Interestingly, CAR’TCR-T cells co-expressing CD33-CAR and dNPM1-TCR were developed to dually target acute myeloid leukemia (AML). dNPM1-TCR is a HLA-A2*02:01-restricted dNPM1-TCR against the neoepitope CLAVEEVSL. The aim of the study was to reduce the risk of tumor escape and relapse. In vitro studies with CD33 and dNPM1 simultaneously expressing OCI-AML cell lines demonstrated that CAR’TCR-T cells were more effective in killing the tumor cells compared to CAR-T cells and TCR-T cells applied separately. The in vivo situation was different. NSG mice were intravenously injected with OCI-AML3 cells expressing both targets. After 4 days, effector CAR-T cells or TCR-T cells, CAR-T cells and TCR-T cells (double T), or CAR-T cells, TCR-T cells and CAR’TCR-T cells (Triple-T) were injected into mice. Tumor elimination could only be seen if all different recombinant T cells (Triple-T) were injected into mice [48].

Instead of fusing the variable domains of a T cell receptor to the chimeric antigen receptor, a TCR-like antibody and T cell receptor-mimetic antibody (TCRm) can be fused to mimic the TCR (Figure 1). Two strategies are possible.

(A) Fusion of the TCR-like antibody to the costimulatory domains and CD3ζ domain or fusion of the costimulatory domains to the CD3ε domain. A TCR-like CAR was developed against the MAGE-A4p230-239/HLA-A*02:01 complex. The TAA is frequently expressed in various types of tumors but not in normal tissues other than the placenta and testis. It was shown that a TCR-like CAR with the CD28 transmembrane domain, the CD3ζ and the GITR (glucocorticoid-induced tumor necrosis factor receptor-(TNFR)-related receptor domain could significantly suppress the growth of MAGE-A4+ HLA-A*02:01+ tumors in an immunocompromised mouse model [49]. Interestingly, the intracellular domains of CD28 or 4-1BB did not significantly suppress the growth of MAGE-A4+ HLA-A*02:01+ tumors.
(B) Fusion of the TCR-like antibody to the CD3ε domain. This leads to the assembly of the two endogenous CD3ζ domains and the CD3γ and CD3δ domains with two CD3ε domains fused to the TCR-like antibody together with the endogenous TCR domains (Vα, Vß and Cα, Cß) expressed on the TCR-like CAR-T cell. The assembled TCR comprises all TCR domains of a native TCR [50]. The efficiency of the TCR-like CAR-T cell was demonstrated by targeting the extra domain B (EDB)-fibronectin. One construct with the costimulatory domain CD28 demonstrated the efficient inhibition of U87MG glioblastoma tumor cell growth in a xenograft tumor mouse model. Interestingly, the recombinant TCR-like CAR-T cells showed better results as a CAR-T cell construct with the TCR-like antibody and the CD3ζ and CD28 or 4-1BB costimulatory domain.

### 2.3. T-CARs

Furthermore, TCR-like antibodies in the scFv format were fused to the constant domains Cα or Cβ of the TCR as a heterodimer (T-CAR) or two scFvs fused to Cα and Cβ, respectively. The transfection of the T-CARs led to the expression of a TCR with all CD3 subunits of the endogenous TCR [18]. Among the T-CAR formats with human C-domains, a bicystronic VH-VH-Cα/VL-VL-Cβ construct led to the highest CD3ε signals. Here, the VH and VL domains were arranged in tandem on the TCR Cα/Cβ-chains. The signaling of the assembled TCR is similar to the signaling of a native TCR (see STARS) (Table 1), (Figure 2). Interestingly, vaccination of tumor-bearing BALB/c mice lymphodepleted by total body irradiation (TBI) and transduced with specific T CAR-T cells and mRNA tumor antigen embedded in lipoplexes enhanced T cell proliferation and tumor cell killing compared to mice without vaccination. This was demonstrated with CLDN18.2+ CT26 tumors (colon carcinoma cell line CT26 expressing tumor-associated target antigen of the Claudin family) and CLDN18.2 mRNA lipoplexes.

### 2.4. STARs

A recently reported synthetic T cell receptor and antigen receptor (STAR) represents a double-chain chimeric receptor, with variable regions from the anti-epidermal growth factor receptor (EGFR) monoclonal antibody Cetuximab and constant regions of the TCR that engage the endogenous CD3 signaling machinery [19] (Figure 1 and Figure 2). This signaling pathway demonstrated a strong and sensitive response compared to CAR signaling, and induced transcriptional changes observed in physiological TCR signaling (Table 1). TCR-engineered T cells recognize intracellular tumor antigens presented by MHC molecules and engage CD3 signaling. This resulted in tumor remission in solid tumors. TCRαβ heterodimers in STAR T cells do not contain their own signaling domains and interact with endogenous CD3εγ, εδ and ζ subunits. These subunits contain ten immunoreceptor tyrosine-rich activation motifs (ITAMs) in contrast to conventional CARs with only three ITAMs. When phosphorylated ITAMs initiate further T cell signaling, it is suggested to result in much stronger and more widespread T cell activation with a greater ITAM multiplicity [19,51]. This could result in superior signaling properties of TCRs compared to CARs, and the physiologic signal transduction machinery of native TCR complexes may provide better antitumor efficacy as observed with STAR cells. Constant regions of the TCR in STAR receptors are ligated with variable regions of heavy and light chains of an antibody. This may transmit high affinity and specificity of the parental antibody and signaling capacity, as in native TCR complexes, into chimeric STAR receptors and thus have comparably higher binding affinity and peptide–MHC stability compared to CAR-T cells (Table 1). The conformation of STARs recapitulates the native TCR structure similar to T CARs [18]. T CARs provide a strategy to improve the antitumor efficacy of T cells against refractory solid tumors like STAR T cells. However, in multiple solid tumor models, STAR T cells resulted in superior or equipotent antitumor effects compared to CAR-T cells without the reported relevant toxicity [19].

### 2.5. Single-Variable-Domain TCR (Svd TCR)

A single-variable-domain TCR (svd TCR) comprising a surrogate TCR Cα, a TCR Cß and a TCR-like scFv has been constructed. The binding of the svd TCR to a neoantigen peptide was realized by the fusion of a TCR-like antibody to the TCR Cß domain. As an example, svd TCRs against the NY-ESO-1 9 V variant peptide (SLLMWITQV) or the MAGE-A3 peptide (FLWGPRALV) in complex with the HLA-A*02-01 allele were constructed. Furthermore, bispecific TCR constructs were generated, containing a surrogate Cα TCR chain and a Cβ TCR chain to which the two scFvs against HLA-A*02-01 allele and MAGE-A3 peptide were coupled in tandem via a (G4S)3GG fexible linker in both orientations. All constructs were able to recognize the antigen peptides [52].

### 2.6. Delivery of mRNA with Lipid Nanoparticles

This strategy has accelerated the treatment of various disorders, including infectious diseases, cancer, and inherited diseases. mRNA-based cancer immunotherapies are very promising due to their high efficiency, pharmacokinetics, safety, and cost-effectiveness and are an attractive alternative to viral gene therapy and protein therapeutics [53,54]. The in vivo transfection of circulating T cells with therapeutic TCR mRNA, TCR-like antibody mRNA, CAR mRNA, TCR-like CAR mRNA, STARS mRNA, T_CARs mRNA and bispecific antibodies mRNA opens new immune-therapeutic opportunities. It has been demonstrated that reprogrammed T cells with CARs or TCRs after in vivo transfection with mRNA/lipid nanoparticles were similarly effective in eliminating tumors in mice compared to conventionally and adoptively transferred T cells [55,56]. This strategy can be used as an alternative to the complex and expensive procedure of retargeting CARs, TCR-engineered T cells, TCR-CARs, TCR-like CARs, STARs and T-CARs to patient tumor cells. Unfortunately, nanoparticles often demonstrate insufficient retention time and low transfection efficiency. To circumvent this disadvantage and to treat B-cell malignancies, an implantable scaffold was recently developed, which releases CD19-specific CAR-T cells generated after retroviral transduction into the bloodstream [57]. Another approach demonstrates the efficient and specific retargeting of CD4 and CD8 T cells in humanized mouse models with lentiviral vector particles and an incorporated chimeric antigen receptor [58]. In vivo transfection with mRNA/lipid nanoparticles seems to be also a very attractive alternative method for the in vivo delivery of intrabodies into tumor cells, which has been mostly performed until now with lentivirus and adenovirus in xenograft tumor mouse models and in vitro [3]. Specific targeting with mRNA/lipid nanoparticles remains crucial for specific immunotherapy. Antibodies or peptide ligands can be fused to lipid nanoparticles [59,60,61], transporting the nanoparticles to the tumor cells. Recently, inhaled mRNA nanoparticles dual-targeting cancer cells and macrophages in the lung for effective transformation have been described [62].

### 2.7. TCR-like Antibodies

To overcome difficult protein engineering approaches to stabilize and solubilize TCR molecules, TCR-like antibodies have been developed (Figure 1). These can be selected by the biopanning of an antibody phage display library with the soluble peptide major histocompatibility complex (MHC) class I complex (pMHC), the full tetrameric pMHC or by the immunization of mice using the recombinant pMHC molecule or pMHC-expressing cells, followed by hybridoma generation, B cell cloning or chip-based single-cell analysis [63]. IgG TCR-like antibodies have been developed to target MHC/TAA (tumor-associated antigen) peptide and MHC/neopeptide complexes through antibody-dependent cellular cytotoxicity (ADCC), antibody-dependent cellular phagocytosis (ADCP) or complement-dependent-cytotoxicity (CDC) [2].

In addition, engineering bispecific monoclonal antibodies, immunotoxins and antibody-drug conjugates (ADCs) with TCR-like antibodies has been successfully performed [2]. Several T cell receptor-mimetic antibodies (TCRms) demonstrated therapeutic effects in xenograft mouse models [64]. The advantage of IgG TCRm antibodies is their stability compared to soluble TCRs [65] (Table 1).

In contrast to TCRs, TCRm antibodies demonstrate more cross-reactivity through binding to hotspots on the HLA/peptide complex surface, whereas the binding of the T cell receptor is dispersed over multiple residues [66]. In general, soluble TCRs must be stabilized and affinity maturated [4]. Stabilization can be introduced by protein engineering, and affinity maturation can be performed by directed evolution. Large, fully or semi-randomized libraries of TCRs displayed on mammalian cells, yeast or phage are used with soluble HLA/peptide complexes as targets [65].

### 2.8. Bispecific Antibodies

Bispecific T cell-engaging proteins comprise two different scFvs covalently linked through a peptide linker. One scFv binds as a TCR mimic to the neopeptide/MHC on tumor cells, whereas the other binds to a member of the CD3 complex. Alternatively, soluble TCRs can be fused to an anti-CD3 scFv to create bispecific proteins termed “immune-mobilizing monoclonal TCRs against cancer” (ImmTAC) and “T cell engaging receptor” (TCER). These promising molecules are now tested in clinical phase 1/2 [67,68] Figure 1.

Two bispecific antibodies comprising an anti-CD3- and TCRm-binding domain against neopeptide/HLA complexes have been successfully tested in xenograft mouse tumor models. One bispecific single-chain diabody construct constitutes an antibody binding domain against CD3 and p53 mutation R172H complexed with HLA2 [69]. The other bispecific single-chain diabody binds to CD3 and RAS-mutated neuropeptides RAS G12V and Q61H/L/R presented on HLA-A3 and HLA-A1 [70]. Both antibodies were able to recognize tumor cells, activate T cells and eliminate the tumor cells in mice.

Tebentafusp is a bispecific antibody (soluble TCR x CD3) and has been approved for the treatment of HLA-A*02:01-positive adult patients suffering from unresectable metastatic uveal melanoma based on a randomized, open-label, multicenter, phase 3 clinical study. Referring to toxicity skin reactions, elevated liver enzymes and embryonic fetal toxicity has been observed [71].

### 2.9. Intrabodies

Intrabodies can image, track and reconstitute intracellular proteins [72]. Intrabodies have been successfully applied to inhibit tumor growth in xenograft tumor mouse models, and several intrabodies have been used to target members of the Ras gene family [3]. Nevertheless, ineffective cell-specific gene delivery has limited the wide-scale application of intrabodies as cancer therapeutics. A new strategy based on delivering intrabody mRNA with lipid nanoparticles may be more advantageous and will pave the way to bring established intrabodies into the clinic. This fascinating delivery strategy has expedited the clinical translation of mRNA therapies for various disorders, including infectious diseases, cancers and inherited diseases [53]. So far, non-replicating mRNAs are mostly investigated in clinical trials for cancer treatment. Recently it was demonstrated that the transfection of the mRNA of an anti-tau scFv and anti-tau IgG embedded into nanoparticles could target tau in the cytoplasm [73]. This demonstrates that the delivery of intrabody mRNA could be utilized in therapeutic human studies.

In contrast to intrabodies recognizing a neutralizing epitope of a neoantigen, so far demonstrating no off-target effects, TCR-like antibodies often demonstrate off-target effects, (Table 1). The reason is that TCR-like antibodies can cross-react with HLA molecules, presenting related peptides besides the desired one, which can result in severe organ toxicities. This was recently shown with ESK1, a TCR-like antibody recognizing wild-type intracellular Wilms Tumor Onco-Protein-1 (WT-1) pMHC. The off-target activity of ESK1 leads to the killing of liver spheroids [74]. Furthermore, cytosolic and nuclear intrabodies neutralizing neoantigen functions can be selected by biopanning with camelid, shark and even human VH and VL phage display antibody repertoires using a purified neoantigen [3,75,76,77] (Figure 2). The selection of TCR-like antibodies is more difficult because biopanning has to be performed with the soluble pMHC or the full tetrameric pMHC [78]. Furthermore, using this strategy, it is uncertain whether it will select a TCR-like antibody against an off-target peptide sequence [74]. To exclude toxic side effects, the TCR-like antibody specificity profile has to be determined. This is possible by testing the specificity directly in human tissues using mass spectrometry [79].

**Table 1:** Generation, advantages and disadvantages of genetically engineered T cells and recombinant antibodies.

Genetically Engineered T Cells	Generation	Advantages	Disadvantages/Adverse Effects
**TCR-engineered** **T cells**	Sorting and expansion of neo-epitope-reactive tumor infiltrated T cells [28]. Sorting neoantigen positive single T cells from the peripheral blood of healthy donors [21,29,30]. Transfection and expansion of patient T cells transfected with recombinant TCR [31].	TCRs have not to be affinity maturated if neoantigens have to be targeted.	Autologous T cells have to be prepared for each patient applying genetically engineered T cells Interference of endogenous TCR signaling with signaling of recombinant TCR (rTCR) signaling. Heterogeneous mixing of the rTCR α and β chains with their endogenous counterparts. Insufficient rTCR expression and inefficient potency. Dosing and potential long-term toxicity can also be difficult to control. Most neoantigens are passenger mutations, increasing the risk of selection of antigen-negative variants. Targeting hot spot mutations, MHC restriction limits clinical applicability of specific TCRs. Targeting strategies applicable to tumors with high mutational burden (melanoma and lung cancer).
**CARs**	Transduction of the CAR gene into patients T cells [34].	Lack of MHC restriction enables applicability to patients expressing surface TAAs regardless of MHC allele expression. Efficient depletion of hematologic cancer. Off-target ‘bystander’ killing of antigen-negative tumor cells.	Risk of toxicity because of low-level expression of TAAs on normal tissues. High TAA density is required for optimal efficiency. Induction of a cytokine release syndrome. Induction of an immune effector cell-associated neurotoxicity syndrome. No efficient targeting of solid tumors due to lack of cell surface TAAs. Tonic signaling leading to T cell dysfunction and cell death.
**TCR CARs**	Transduction of patients T cells with a neoantigen specific TCR-CAR construct [17].	Targeting of solid tumors that express no cell surface TAAs or cell surface neoantigens.	Interference of endogenous TCR signaling with signaling of rTCR signaling. Heterogeneous mixing of the rTCR α and β chains with their endogenous counterparts.
**TCR-like CARs**	Transduction of patient T cells with a TCR-like CAR construct [17].	No interference of endogeneous TCR signaling with TCR-like CAR receptor signaling. High affinity of the TCR-like antibody to the MHC/neopeptide complex compared to TCR.	Crossreactivity greater compared to TCRs.
**STARs/T-CARS**	Transfection of STAR or T-CAR into patient T cells [18,19].	Signaling of STAR/T-CAR resembles signaling of native TCR. No tonic signaling.	So far no disadvantages known.
**Recombinant antibodies**			
**IgG TCR-like antibodies**	TCR-like antibodies can be selected by biopanning of an antibody phage display library or by the hybridoma technique [63].	High stability to the pMHC class I-complex compared to soluble TCR.	Crossreactivity greater compared to TCRs.
**Bispecific antibodies**	Fusion of an anti-CD3 scFv with a TCR-like antibody (scFv) or with a soluble TCR [67].	Off-target ‘bystander’ killing of antigen-negative tumor cells. Cytokine release syndrome lower than induced by CAR T cells.	Induction of a clinically relevant cytokine release.
**Intrabodies**	Selection of neutralizing neoantige-specific intrabodies using camel, shark or human VH or VL phage antibody repertoires [75,76,77].	High specificity to the neutralizing neoantigen epitope compared to TCRs and TCR-like antibodies.	Until now adverse effects unknown. Transfection of intrabody mRNA with lipid nanoparticles could pave the way into routine clinical application.

### 2.10. Clinical Implications

Several tests of overall health and fitness need to be performed before the patient can be considered a candidate for T cell therapy. This differs from other protocols using established antibodies. Also, T cell therapies need the prior administration of a lymphodepleting chemotherapy to reduce the level of white blood cells and increase the infused T cell effectiveness. For this purpose, the patients need to be hospitalized for several days after the infusion of the T cells so that their response can be monitored and potential adverse events may be treated. Moreover, a cytokine release syndrome represents one of the serious adverse events after a T cell therapy, which is characterized by flu-like symptoms such as high fever, fatigue and headache, and can escalate to more serious circulatory disturbances depending on dose-escalating regimens. When a clinically relevant dynamic response in a patient develops, it needs to be treated with immunosuppressants such as the anti-IL-6 antibody tocilizumab. Another serious adverse event that may occur after T cell infusion is an immune effector cell-associated neurotoxicity syndrome, which typically occurs within a week after the T cell infusion and presents with symptoms such as tremors, speech impairment, delirium and confusion. Both adverse events need experience in identifying and managing such neurotoxicities and cytokine- or inflammation-mediated disturbances in the patients. Moreover, the manufacturing process of every individual recombinant T cell therapy currently takes between four and six weeks of time from the day the cells are obtained from leukopheresis. Notably, the overall costs of such T cell therapy are significant and exceed the cost of established antibody therapies.

### 2.11. Therapeutic Limitations

The therapeutic limitations for the efficiency of genetically engineered T cells are complex and are hence the basis for further improvements. Adoptively transferred T cells are supposed to be short-lived in the circulation of many patients, particularly in solid human cancer. Other reasons for their limited efficiency are tumor antigen heterogeneity, impaired T cell trafficking, extravasation to tumor sites and also T cell exhaustion. Furthermore, therapeutic effects are often hampered by the loss of neoantigens due to the loss of allelic MHC, downregulation of MHC molecules, downregulation of neoantigen transcription or defects of the antigen-processing machinery, translational repression, copy number loss, and the modification of the process of neopeptide presentation after posttranslational modification. Moreover, an immunosuppressive tumor microenvironment can inhibit T cell recognition and activation [80].

Impaired T cell infiltration to the tumor site may be increased by chemokine and cytokine receptors and the corresponding soluble molecules, which can be expressed by adoptively transferred T cells or co-delivered with the administration of the T cells [80]. Interestingly, the co-expression of tumor necrosis factor (TNF) superfamily member 14 (LIGHT) in CAR-T cells reconstituted a high immune-infiltrated tumor microenvironment and also improved CCL19 and CCL21 expression by surrounding cells, as shown in an immunodeficient NSG prostatic tumor mouse model [81].

To mitigate neoantigen loss, it was more recently shown that T cells transfected with bispecific tandem CARs fused with a linker and bicystronic or tricystronic engineered CARs targeting multiple antigens could prevent the risk of tumor antigen escape [5]. In contrast to expressing different engineered CARs on a single T cell, cancer patients could be transfected with a combination of different T cells expressing unique, engineered TCRs, each recognizing a single neoantigen peptide/MHC estimated previously using a personalized library of soluble predicted neoantigen–HLA capture reagents [31].

Moreover, the use of immune checkpoint inhibitors like anti-PD-1 and anti-PD-L1 monoclonal antibodies has been successfully applied in different cancers like liver cancer and lung cancer, melanoma and Hodgkin’s lymphoma [82,83,84]. An immune checkpoint blockade (ICB) with monoclonal antibodies could reactivate CD8^+^ T cells in the periphery and microenvironment. Interestingly, in ICB-responsive cancer patients, the progenitors of exhausted T cells (Tpex) within the tumor microenvironment are likely the cell population revived by ICB therapies, whereas T cells in the periphery possess a more activated cellular state [85]. Alternatively, the knockout of T cell PD-1 expression can be performed with CRISPR/Cas9. Adoptive T cell transfer, in which PD-1 was genetically disrupted, was performed in 12 patients suffering from metastatic non-small cell lung cancer (NSCLC) and 17 patients with advanced esophageal squamous cell carcinoma (ESCC). Approximately 18% of the NSCLC and 35% of the ESCC patients demonstrated stable disease [86].

CRISPR–Cas9 knockout of endogenous TCRαβ can enhance the transgenic T cell receptor expression and functions during the TCR gene therapy and prevent graft versus host rejection. In recent clinical trials using NY-ESO-1 TCR-T cells, endogenous TCR and PD-1 were knocked out using the CRISPR-Cas9 genome editing tool. Although no clinical response was observed among the three treated patients, the in vivo persistence of the CRISPR-engineered T cells was increased (36 weeks versus 1 week) compared to other trials studying NY-ESO-1 [87,88]. While showing promising results, the prevalence of NY-ESO-1 expression is still limited in metastatic cancers, and its tumor expression is often heterogeneous [89].

It was recently shown that the knockout of the polibrumo-associated BAF (PBAF) chromatin remodeling complex in T cells favors responses to the PD1 pathway blockage in tumor melanoma mouse models. PBAF is a form of the remodeling SWI/SNF complex and induces T cell factor (TCF+) progenitor T cells to a more exhausted state. *P14* cells were obtained from mice, in which PBAF was knocked out with CRISPR/Cas9 after mice were inoculated with B16GP33 melanoma cells. The treatment of these mice with anti-PD1 antibodies showed a significant decrease in melanoma tumor size, and four of ten animals survived until day 35 compared to no mice when receiving only control T cells. In contrast, mice receiving only the recombinant T cells without blocking antibodies showed a lower decrease in melanoma tumor size [90].

Tumor endothelial cells (ECs) are characterized by diverse and dynamic states, including abnormally proliferating, senescent, transdifferentiated and immune-modulatory ECs [91]. Many improvements to target tumor angiogenesis have been described. The combination of anti-angiogenesis inhibitors with immune checkpoint inhibitors has been successfully established [91]. For example, a combination of the anti-VEGF antibody avastin with atezolizumab (anti-PD1-L1 blocking antibody) was more effective than treatment with the protein kinase inhibitor sorafenib alone in advanced hepatocellular carcinoma [92]. The treatment of patients with advanced renal cell carcinoma with the VEGFR inhibitor axitinib and pembrolizumab (a PD-1-blocking antibody) demonstrated better results than treatment with sunitinib alone [93]. Other inhibitors, including axitinib, apatinib, lenvatinib, cabozantinib, pazopanib and regorafenib, block VEGFRs and other tyrosine kinases, including PDGFRs, KIT, TIE2, FGFRs and cMET. Approval has been obtained for colorectal, renal cancer and hepatocellular carcinoma, often applied in the advanced or metastatic setting [94].

Targeting the extracellular matrix for cancer treatment has included studying the extra domain B (EDB)-fibronectin, a protein with restricted expression pattern in the extracellular matrix of tumor neovasculature. EDB-fibronectin TCR-like CARS prevented the growth of new blood vessel formation in the tumor environment associated with the inhibition of tumor growth [50]. Furthermore, several other strategies harnessing the tumor microenvironment to boost adoptive T cell therapy have been performed with CAR-T cells [95]. CAR-T cells were generated, expressing chemokine receptors, synthetic receptors targeting suppressive cytokines, inhibitory signals and pro-inflammatory cytokines.

Some of the most promising strategies are those using genetically engineered T cells that secrete antibodies, enzymes and immunostimulatory molecules to specifically modulate the tumor environment [96]. Another attractive approach represents natural killer cells expressing recombinant TCRs (TCR-NK cells) [97]. Using a melanoma xenograft mouse model, it was demonstrated that NK-TCR cells increased the survival of treated mice compared to mice treated with NK cells without melanoma-specific TCR. In a phase 1/2 trial, NK cells derived from cord blood expressing anti-CD19 CAR and interleukin (IL-15) were tested in 37 patients with CD19^+^ B cell malignancies. The 1-year overall survival and progression-free survival were 68% and 32%, respectively. Most responses were complete responses, with 1-year cumulative complete response rates of 83%, for low-grade non-Hodgin lymphoma (NHL), 50% for chronic lymphocytic leukemia (CLL) and 29% for diffuse large B-cell lymphoma (DLBCL) [98].

## 3. Conclusions

Over the last seven years, the U.S. Food and Drug Administration (FDA) and the European Medicines Agency (EMA) have approved six CAR-T cell therapies for use in patients with B cell malignancies and multiple myeloma. A limited number of clinical trials are currently underway to investigate recombinant T cell therapies in patients with solid tumors of the lung, breast, pancreas and ovaries, as well as melanoma, demonstrating promising results. However, it is still too early to draw conclusions from the small number of participating patients and limited imaging and biopsy data in these studies.

Despite new opportunities of such engineered immune cell therapies and promising response rates, certain challenges remain, including the significant work that goes into preparing the patients, the time-consuming preparation of recombinant T cell therapies and the possible adverse events after their administration.

The targeting of neoantigens with recombinant T cells comprises the identification of patients’ MHC/neopeptide complex and cloning, expression, testing and transduction of the newly identified TCR in patients’ T cells, whereas antibodies must be selected by phage display and tested with the newly identified pMHC or neoantigen. Nevertheless, the signaling of recombinant TCRs in TCR-T cells seems to be comparable to the signaling of native TCR. They are more sensitive, reacting to a small amount of antigen, and demonstrate a more gradual signal transmission compared to T cells with a CAR.

Only 1% of the MHC-binding peptides have been reported to be recognized by patient T cells. Theoretically, a growing tumor burden leads to an increase in the amount of different CD8^+^ T cells that might recognize a growing number of neopeptide/MHCs and subsequently become activated. Generally, the identification of personalized neopeptides in cancers with a low tumor burden demands great efforts to identify suitable neopeptide/MHCs. This could be achieved by combining tumor DNA/RNA sequencing and mass spectrometry proteomics [99]. Recently, a subset of CD8^+^ T cells, termed CD8-fit T cells, have been identified within axicabtagene ciloleucel (axi-cel) anti-CD19 CAR-T cells [100]. CD8-fit T cells are present and persist in individuals even after treatment with CAR-T cells and are endowed with migratory capacity and serial killer activity. The analysis of the T cell infusion product by using scRNA-seq analysis, confocal microscopy and timelapse imaging microscopy detected CD8-fit cells with effective therapeutic potential. Therefore, the characterization of such cells will be of importance for predictable and durable clinical therapies.

Nevertheless, antibody-based cancer therapy against intracellular neopeptides appears to be easier to perform as a T cell therapy. In particular, an intrabody therapy, with its easy selection and high specificity, seems to be very promising, comparably safe and less expensive. Recently, two intrabodies were selected, blocking TLR2 and TLR9 and inhibiting ex vivo inflammation-mediated pancreatic cancer cell growth [101].
